# Prevalence of Non-Communicable Diseases and Its Associated Factors Among Urban Elderly of Six Indian States

**DOI:** 10.7759/cureus.30123

**Published:** 2022-10-10

**Authors:** Meenakshi Chobe, Shivaji Chobe, Sonal Dayama, Amit Singh, Kashinath Metri, Jagannadha R Basa, Nagaratna Raghuram

**Affiliations:** 1 Yogic Sciences, Swami Vivekananda Yoga Anusandhana Samsthana, Bangalore, IND; 2 Preventive Medicine, Swami Vivekananda Yoga Anusandhana Samsthana, Bangalore, IND; 3 Integrative Medicine, RESET Tech Global Pvt. Ltd., Mumbai, IND; 4 Public Health, All India Institute of Medical Sciences, Raipur, IND; 5 Yoga, Central University of Rajasthan, Rajasthan, IND; 6 School of Engineering, International School of Engineering, Hyderabad, IND

**Keywords:** urban health determinants, obesity, sleep, joint pain, diabetes mellitus, hypertension, multimorbidity, geriatrics, non-communicable diseases

## Abstract

Aims

The aim of this study is to investigate the prevalence, impact of health determinants on non-communicable diseases (NCDs), and multimorbidity among urban elderly in India.

Methods

This is a cross-sectional study involving a total of 1,671 (870 male and 801 female) respondents aged 60-80 years. Multistage sampling was used for the recruitment of the participants. A total of 12 sample areas from 12 cities of six southern states of south India were selected. Through survey form, information regarding demographic characteristics, health-influencing lifestyle factors, and history of nine NCDs was collected.

Results

The mean age of participants was 68.5 ± 6.01 years.. The prevalence of hypertension was 40.4%, followed by diabetes (31.2%), arthritis (22.1%), sensory impairment (10.1%), heart diseases (7.8%), and dyslipidemia (7.0%). 74.1% of participants had at least one morbidity, and 40.0% of people had multimorbidity. Being overweight is the highest risk health determinant for hypertension, diabetes, heart disease, high cholesterol, stroke, and joint pain. Obese people have 64% more risk of hypertension than people with normal BMI. People with disturbed sleep have increased risk of hypertension, high cholesterol, and joint pain by more than 80% compared to people with proper sleep. Among the modifiable health determinants of obesity, disturbed sleep, constipation, and physical activity up to 30 minutes were positively associated with multimorbidity. Those in the age group of 70 to 80 years have a high risk for NCDs and multimorbidity compared to those in the age group of 60 to 70 years.

Conclusions

A healthy lifestyle is necessary to reduce the burden of NCDs among the elderly. Developing holistic health policies seems an urgent need.

## Introduction

The world population is rapidly aging due to increased longevity, decreased fertility, and mortality rates [1]. As per World Population Prospects, one in 11 individuals was above 65 years in 2019, and by 2050, one in six people will be above 65 years of age [2]. The elderly are at higher risk for multiple health challenges. Non-communicable diseases (NCDs) are highly prevalent in later life. NCDs have become a global health agenda because they cause global morbidity, disability, and death in later life [3]. Degenerative aging processes, environmental, social, and unhealthy lifestyles are risk factors for diseases in old age.

Along with aging, behavioral factors such as tobacco use, physical inactivity, excess consumption of alcohol, and an unhealthy diet further increase the risk of NCDs and mortality due to NCDs [4,5]. Globally every year, 71% of deaths are due to NCDs. Three-fourths of these deaths occur in low- and middle-income countries [6]. The presence of two or more chronic diseases called multimorbidity is also increasing worldwide [7]. Low- and middle-income countries like India face multiple health challenges arising from NCDs and an increasingly elderly population. World Health Organization gives importance to healthy aging [8].

People living in urban areas are increasing worldwide [9]. It is leading to serious health challenges [10]. Urbanization is directly related to unhealthy behavioral adoption [11,12]. It increases the risk of NCDs [13]. Urban residents are more likely to have multimorbidity [14]. Elderly urban residents are not exceptions to these health challenges [15]. Mortality rates are high among individuals with multimorbidity [16]. Currently, there is a need for more population-based estimates of NCDs among the urban elderly in India. Knowing the current epidemiology of NCDs among the urban elderly will help formulate preventive, promotive, and curative health. With this objective, we undertook a prevalence study among six southern states of India.

## Materials and methods

Study design and setting

**Figure 1:**
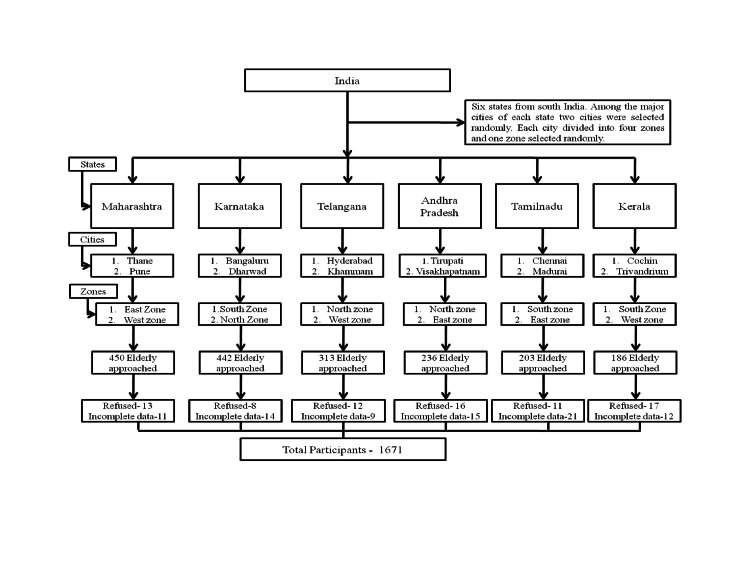
Flow chart of the recruitment of the urban elderly residents

A stratified multistage sampling strategy was chosen for sampling. The study was conducted from October 2018 to September 2019. The study covered urban areas of six states of south India (Kerala, Tamil Nadu, Karnataka, Andhra Pradesh, Telangana, and Maharashtra). From each state, major city names were listed. Out of these, two cities were selected based on a higher proportion of urban population selectively in each state. A total of 12 cities selected were Cochin, Trivandrum, Chennai, Madurai, Bengaluru, Dharwad, Tirupati, Visakhapatnam, Hyderabad, Khammam, Pune, and Thane. The selected city was divided into four geographical zones (East, West, South, North). Furthermore, one geographical zone was selected randomly, and from the selected zone, one residential locality/ward was selected randomly for the survey. Local health workers/influential leaders were contacted for the cooperation and support required during data collection. This helped in decreasing the non-response rate. One household was selected randomly, and after that, subsequent households were interviewed sequentially till approximately 150 elderly were selected from each city based on the overall sample size estimated for the study. In a household where more than one elder person lived, only one was interviewed. Details are shown in Figure 1.

Twelve sample areas from 12 cities in six states were selected. The Institutional Ethics Committee of Swami Vivekananda Yoga Anusandhana Samsthana issued approval (RES/IEC-SVYASA/117/2017). Written informed consent was obtained from each participant before interviewing.

Participants

For participants, inclusion criteria were as follows: (1) people aged between 60 and 80 years, (2) living in urban areas for more than 20 years, and (3) willingness to participate and give written informed consent. The elderly living in households and residents of elderly care homes of the selected locality participated in the study. The survey sample size was calculated using Raosoft Inc. (2004). Keeping the confidence level 99%, the margin of error at 3%, and response distribution at 50%, the elderly population in selected six states was 38.7 million. A total of 1,843 was the calculated sample size. After contacting the participants, they were screened at their residence for inclusion. A total of 2,076 elderly were screened, and 1,830 met the inclusion criteria. A total of 1,753 agreed to participate in the survey study, and after the removal of incomplete data, data of 1,671 participants were included in the analysis.

Data collection and measurements

A trained interviewer interviewed all the participants who consented. All the field workers underwent training prior to starting the study. They were trained using a standard protocol, a standard questionnaire, and methods of taking measurements. Participants were interviewed at their residences to reduce the non-responsive bias. The information was collected from the participants for the demographic characteristics, health-influencing lifestyle factors, and history of medical illness through a standard pre-tested questionnaire. As mentioned earlier in Figure 1, missing or incomplete forms were not taken into the final analysis.

Demographic information included gender (male and female), age, marital status (married, never married, divorced, and widowed), residence (living with family, living separate, and elderly care home), education (total years of education categorized into 1 to 5, 6 to 12, and more than 12 years), employment (self-employed, private sector, government sector), present work status (retired, full time working, part-time working, homemaker), and economic status (dependent on family, pension, salary, self-sustainable).

Health-influencing lifestyle factors were assessed by collecting information on diet type (vegetarian or non-vegetarian), kind of physical activity (nil or physical activity-walking, aerobics, yoga), duration of physical activity (less than a half-hour or up to one hour), the total period of physical activity in years, substance abuse (nil or substance abuse such as smoking, alcoholic, tobacco chewing, or any other), substance abuse history (current or past), total years of substance abuse, sleep (normal or disturbed), and bowels clearance (normal, constipation, irregular).

History of a prior diagnosis of hypertension, diabetes mellitus, heart diseases, dyslipidemia, sensory impairment (eyes or ear), stroke, psychiatric illness, arthritis/joint pain, and any other disease was recorded. This information was based on a previously diagnosed illness by a physician. The total duration of illness (in years) and treatment for each disease were also recorded. Height was measured manually by measuring tape in centimeters, and weight was measured by a Belita -1101 mechanical personal weighing machine. The body mass index was calculated using a standard formula for each participant. Multimorbidity was defined as having at least two chronic conditions [17].

Data analysis

Statistical analysis was conducted using the open source software R version 4.03. Descriptive analyses were performed to determine the distribution of demographic and health-influencing factors to calculate the prevalence proportions of different NCDs. In addition, we constructed two sets of models. Stepwise logistic regressions were performed for eight NCDs (hypertension, diabetes mellitus, heart disease, high cholesterol, sensory impairment, stroke, psychiatric illness, joint pain) to assess the strength and direction of the association between NCDs and their potential correlates.

We further performed a multivariate logistic regression model adjusting the odds ratio for modifiable and nonmodifiable factors separately. For each regression analysis, we used appropriate model diagnostics, and the models that we used fit well (p<0.01). The adjusted odds ratio of modifiable and nonmodifiable factors was obtained from the stepwise logistic regression model. This model uses the Akaike information criterion (AIC) to iteratively add and remove the predictors to find the subset of variables in the given data set resulting from the best-performing model, which has a lower prediction error.

Furthermore, we created a dependent variable (number of NCDs) with three clearly defined categories (suffering from no NCD/one NCD/two NCDs) with a standard order (zero, one, and two or more). This analysis aimed to determine the association of the independent variables with the odds of having a higher number of NCDs. To increase analysis efficiency, we built three multivariate step logistic regression models: first, no disease versus one disease; second, no disease versus more than one disease; and third, one disease versus more than one disease.

## Results

Of 1,671 urban elderly, 870 (52.1%) were male and 801 (47.9%) were female. The details of health determinants are as follows. Overall, 54.3% were vegetarian and 45.7% were non-vegetarian. Also, 19.7% were not doing any physical activity. Most (80.3%) were engaged in physical activity like walking, aerobics, or yoga. Of these, participants doing physical activity for less than half an hour were 35.6% and those for up to one hour were 44.7%. One-fifth (20.7%) used smoking, alcohol, tobacco chewing, or other substance. Of the substance users, 9.0% are still taking these drugs, and 11.7% left the substance use in the past. One-fifth of people reported that they had disturbed sleep (20.8%) and constipation (19.7 %). Nearly half (47.3%) of participants were obese (body mass index ≥ 25), and 2.6% of participants were underweight (body mass index < 18.5). Details are given in Table 1.

**Table 1:** Self-reported prevalence of NCDs along with demographic factors and health determinants among elderly NCD, non-communicable disease

Health-influencing factors		Total (%)	Hypertension	Diabetes mellitus	Heart disease	Dyslipidemia	Sensory impairment	Stroke	Psychiatric illness	Joint pain	Any other illness
			n (%)	n (%)	n (%)	n (%)	n (%)	n (%)	n (%)	n (%)	n (%)
Gender	Male	870 (52.1)	309 (35.5)	270 (31)	88 (10.1)	51 (5.9)	89 (10.2)	18 (2.1)	4 (0.5)	129 (14.8)	140 (16.1)
	Female	801 (47.9)	366 (45.7)	252 (31.5)	43 (5.4)	66 (8.2)	79 (9.9)	22 (2.7)	7 (0.9)	240 (30)	144 (18)
Age range, years	60 to 65	614 (36.7)	216 (35.2)	186 (30.3)	28 (4.6)	35 (5.7)	36 (5.9)	8 (1.3)	5 (0.8)	112 (18.2)	95 (15.5)
	66 to 70	480 (28.7)	200 (41.7)	145 (30.2)	41 (8.5)	30 (6.3)	30 (6.3)	11 (2.3)	1 (0.2)	107 (22.3)	93 (19.4)
	71 to 75	365 (21.8)	158 (43.3)	125 (34.2)	31 (8.5)	31 (8.5)	61 (16.7)	10 (2.7)	3 (0.8)	96 (26.3)	69 (18.9)
	76 to 80	212 (12.7)	101 (47.6)	66 (31.1)	31 (14.6)	21 (9.9)	41 (19.3)	11 (5.2)	2 (0.9)	54 (25.5)	27 (12.7)
Marital status	Married	1307 (78.2)	481 (36.8)	402 (30.8)	97 (7.4)	85 (6.5)	121 (9.3)	30 (2.3)	6 (0.5)	248 (19)	213 (16.3)
	Never married	30 (1.8)	15 (50)	7 (23.3)	2 (6.7)	3 (10)	2 (6.7)	0 (0)	1 (3.3)	13 (43.3)	7 (23.3)
	Divorced	11 (0.7)	8 (72.7)	5 (45.5)	2 (18.2)	1 (9.1)	0 (0)	1 (9.1)	0 (0)	5 (45.5)	1 (9.1)
	Widowed	323 (19.3)	171 (52.9)	108 (33.4)	30 (9.3)	28 (8.7)	45 (13.9)	9 (2.8)	4 (1.2)	103 (31.9)	63 (19.5)
Residence	With family	1197 (71.6)	485 (40.5)	403 (33.7)	102 (8.5)	84 (7)	104 (8.7)	29 (2.4)	9 (0.8)	238 (19.9)	173 (14.5)
	Separately	107 (6.4)	42 (39.3)	30 (28)	8 (7.5)	5 (4.7)	12 (11.2)	1 (0.9)	0 (0)	32 (29.9)	21 (19.6)
	Elderly care home	367 (22)	148 (40.3)	89 (24.3)	21 (5.7)	28 (7.6)	52 (14.2)	10 (2.7)	2 (0.5)	99 (27)	90 (24.5)
Education	1 to 5 years	116 (6.9)	41 (35.3)	23 (19.8)	6 (5.2)	4 (3.4)	14 (12.1)	3 (2.6)	1 (0.9)	29 (25)	27 (23.3)
	6 to 12 years	672 (40.2)	284 (42.3)	213 (31.7)	47 (7)	43 (6.4)	73 (10.9)	18 (2.7)	6 (0.9)	165 (24.6)	125 (18.6)
	Above 12 years	838 (50.1)	329 (39.3)	273 (32.6)	77 (9.2)	68 (8.1)	75 (8.9)	19 (2.3)	4 (0.5)	160 (19.1)	127 (15.2)
	Uneducated	45 (2.7)	21 (46.7)	13 (28.9)	1 (2.2)	2 (4.4)	6 (13.3)	0 (0)	0 (0)	15 (33.3)	5 (11.1)
Employment type	Self-employed	388 (23.2)	131 (33.8)	100 (25.8)	33 (8.5)	20 (5.2)	37 (9.5)	9 (2.3)	1 (0.3)	57 (14.7)	76 (19.6)
	Government	510 (30.5)	214 (42)	170 (33.3)	49 (9.6)	43 (8.4)	49 (9.6)	12 (2.4)	5 (1)	111 (21.8)	85 (16.7)
	Private sector	366 (21.9)	130 (35.5)	118 (32.2)	27 (7.4)	22 (6)	42 (11.5)	9 (2.5)	1 (0.3)	74 (20.2)	49 (13.4)
	Housewife	407 (24.4)	200 (49.1)	134 (32.9)	22 (5.4)	32 (7.9)	40 (9.8)	10 (2.5)	4 (1)	127 (31.2)	74 (18.2)
Present work status	Retired	932 (55.8)	378 (40.6)	290 (31.1)	86 (9.2)	64 (6.9)	105 (11.3)	25 (2.7)	6 (0.6)	192 (20.6)	159 (17.1)
	Working full time	175 (10.5)	51 (29.1)	57 (32.6)	11 (6.3)	12 (6.9)	12 (6.9)	0 (0)	0 (0)	23 (13.1)	27 (15.4)
	Working part time	135 (8.1)	40 (29.6)	34 (25.2)	10 (7.4)	10 (7.4)	12 (8.9)	5 (3.7)	1 (0.7)	20 (14.8)	22 (16.3)
	Housewife	429 (25.7)	206 (48)	141 (32.9)	24 (5.6)	31 (7.2)	39 (9.1)	10 (2.3)	4 (0.9)	134 (31.2)	76 (17.7)
Economic status	Dependent on family	541 (32.4)	245 (45.3)	162 (29.9)	37 (6.8)	34 (6.3)	65 (12)	17 (3.1)	2 (0.4)	161 (29.8)	108 (20)
	Pension	780 (46.7)	305 (39.1)	249 (31.9)	63 (8.1)	62 (7.9)	80 (10.3)	17 (2.2)	9 (1.2)	155 (19.9)	110 (14.1)
	Salary	62 (3.7)	16 (25.8)	19 (30.6)	7 (11.3)	5 (8.1)	3 (4.8)	1 (1.6)	0 (0)	5 (8.1)	11 (17.7)
	Self-sustainable	288 (17.2)	109 (37.8)	92 (31.9)	24 (8.3)	16 (5.6)	20 (6.9)	5 (1.7)	0 (0)	48 (16.7)	55 (19.1)
Diet type	Vegetarian	907 (54.3)	363 (40)	287 (31.6)	66 (7.3)	51 (5.6)	83 (9.2)	18 (2)	4 (0.4)	170 (18.7)	154 (17)
	Non-vegetarian	764 (45.7)	312 (40.8)	235 (30.8)	65 (8.5)	66 (8.6)	85 (11.1)	22 (2.9)	7 (0.9)	199 (26)	130 (17)
Walking	No	402 (24.1)	166 (41.3)	135 (33.6)	28 (7)	18 (4.5)	46 (11.4)	15 (3.7)	1 (0.2)	125 (31.1)	89 (22.1)
	Yes	1269 (75.9)	509 (40.1)	387 (30.5)	103 (8.1)	99 (7.8)	122 (9.6)	25 (2)	10 (0.8)	244 (19.2)	195 (15.4)
Aerobics	No	1651 (98.8)	669 (40.5)	514 (31.1)	129 (7.8)	113 (6.8)	168 (10.2)	39 (2.4)	11 (0.7)	364 (22)	277 (16.8)
	Yes	20 (1.2)	6 (30)	8 (40)	2 (10)	4 (20)	0 (0)	1 (5)	0 (0)	5 (25)	7 (35)
Yoga	No	1343 (80.4)	556 (41.4)	422 (31.4)	108 (8)	87 (6.5)	137 (10.2)	33 (2.5)	7 (0.5)	318 (23.7)	232 (17.3)
	Yes	328 (19.6)	119 (36.3)	100 (30.5)	23 (7)	30 (9.1)	31 (9.5)	7 (2.1)	4 (1.2)	51 (15.5)	52 (15.9)
Physical activity duration	Nil	329 (19.7)	149 (45.3)	114 (34.7)	24 (7.3)	15 (4.6)	39 (11.9)	12 (3.6)	1 (0.3)	110 (33.4)	73 (22.2)
	Less than 30 min.	595 (35.6)	237 (39.8)	172 (28.9)	48 (8.1)	46 (7.7)	60 (10.1)	14 (2.4)	5 (0.8)	151 (25.4)	99 (16.6)
	Up to 1 hour	747 (44.7)	289 (38.7)	236 (31.6)	59 (7.9)	56 (7.5)	69 (9.2)	14 (1.9)	5 (0.7)	108 (14.5)	112 (15)
Smoking	No	1456 (87.1)	585 (40.2)	462 (31.7)	105 (7.2)	101 (6.9)	144 (9.9)	36 (2.5)	9 (0.6)	327 (22.5)	240 (16.5)
	Yes	215 (12.9)	90 (41.9)	60 (27.9)	26 (12.1)	16 (7.4)	24 (11.2)	4 (1.9)	2 (0.9)	42 (19.5)	44 (20.5)
Alcoholic	No	1500 (89.8)	596 (39.7)	470 (31.3)	109 (7.3)	105 (7)	155 (10.3)	39 (2.6)	10 (0.7)	338 (22.5)	256 (17.1)
	Yes	171 (10.2)	79 (46.2)	52 (30.4)	22 (12.9)	12 (7)	13 (7.6)	1 (0.6)	1 (0.6)	31 (18.1)	28 (16.4)
Tobacco chewing	No	1629 (97.5)	656 (40.3)	508 (31.2)	128 (7.9)	116 (7.1)	164 (10.1)	40 (2.5)	11 (0.7)	360 (22.1)	274 (16.8)
	Yes	42 (2.5)	19 (45.2)	14 (33.3)	3 (7.1)	1 (2.4)	4 (9.5)	0 (0)	0 (0)	9 (21.4)	10 (23.8)
Any other substance habit	No	1655 (99)	667 (40.3)	516 (31.2)	130 (7.9)	117 (7.1)	165 (10)	40 (2.4)	11 (0.7)	361 (21.8)	279 (16.9)
	Yes	16 (1)	8 (50)	6 (37.5)	1 (6.3)	0 (0)	3 (18.8)	0 (0)	0 (0)	8 (50)	5 (31.3)
Substance habit status	Nil	1331 (79.7)	537 (40.3)	425 (31.9)	92 (6.9)	92 (6.9)	136 (10.2)	36 (2.7)	8 (0.6)	303 (22.8)	220 (16.5)
	Current	150 (9)	52 (34.7)	39 (26)	14 (9.3)	10 (6.7)	10 (6.7)	0 (0)	0 (0)	23 (15.3)	31 (20.7)
	Past	190 (11.4)	86 (45.3)	58 (30.5)	25 (13.2)	15 (7.9)	22 (11.6)	4 (2.1)	3 (1.6)	43 (22.6)	33 (17.4)
Sleep	Normal	1323 (79.2)	494 (37.3)	402 (30.4)	101 (7.6)	81 (6.1)	123 (9.3)	29 (2.2)	10 (0.8)	253 (19.1)	211 (15.9)
	Disturbed	348 (20.8)	181 (52)	120 (34.5)	30 (8.6)	36 (10.3)	45 (12.9)	11 (3.2)	1 (0.3)	116 (33.3)	73 (21)
Bowel pattern	Normal clearance	1341 (80.3)	526 (39.2)	423 (31.5)	108 (8.1)	88 (6.6)	113 (8.4)	28 (2.1)	7 (0.5)	256 (19.1)	201 (15)
	Constipation	204 (12.2)	97 (47.5)	67 (32.8)	12 (5.9)	17 (8.3)	37 (18.1)	9 (4.4)	4 (2)	77 (37.7)	49 (24)
	Irregular clearance	126 (7.5)	52 (41.3)	32 (25.4)	11 (8.7)	12 (9.5)	18 (14.3)	3 (2.4)	0 (0)	36 (28.6)	34 (27)
BMI range	Underweight	43 (2.6)	11 (25.6)	6 (14)	2 (4.7)	0 (0)	9 (20.9)	1 (2.3)	0 (0)	10 (23.3)	10 (23.3)
	Normal weight	837 (50.1)	296 (35.4)	233 (27.8)	56 (6.7)	51 (6.1)	90 (10.8)	29 (3.5)	6 (0.7)	165 (19.7)	140 (16.7)
	Overweight and obese	791 (47.3)	368 (46.5)	283 (35.8)	73 (9.2)	66 (8.3)	69 (8.7)	10 (1.3)	5 (0.6)	194 (24.5)	134 (16.9)
Total		1671(100)	675 (40.4)	522 (31.2)	131 (7.8)	117 (7)	168 (10.1)	40 (2.4)	11 (0.7)	369 (22.1)	284 (17)

Prevalence

**Figure 2:**
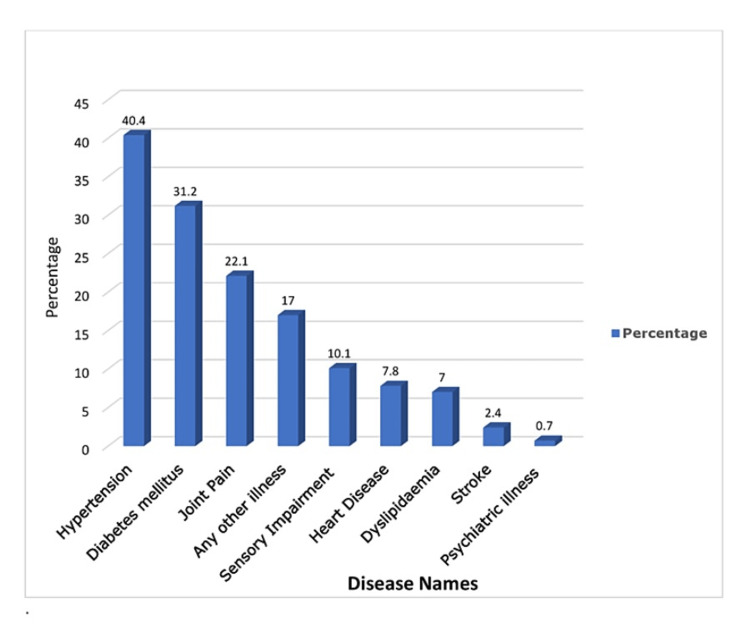
Graph showing non-communicable disease prevalence among the elderly

Table 1 gives the details of nine self-reported NCD prevalence by sociodemographic factors and health determinants among the urban elderly. Among all the reported NCDs, the prevalence of hypertension was higher than in other NCDs. The overall prevalence of hypertension was 40.4%, followed by diabetes (31.2%), arthritis (22.1%), sensory (audio/visual) impairment (10.1%), heart diseases (7.8%), dyslipidemia (7.0%), stroke (2.4%), and psychiatric disorders (0.7%). Overall, 17.0% elderly had some other NCDs (breast cancer, bronchial asthma, hypothyroidism, allergies, renal dysfunction, sarcoidosis, etc.). For details, see Figure 2.

Sociodemographic factorwise, the prevalence of hypertension was higher in females (45%), those aged between 76 and 80 years (47.6%), widowed (52.9%), housewives (49.1%), and economically dependent on family (45.3%). Health determinant factorwise, prevalence of hypertension was higher those who were physically inactive (45.3%), having disturbed sleep (52.0%), having constipation (47.5%), and obese people (46.5%). The prevalence of diabetes was higher among those who were physically inactive (34.7%), those who had disturbed sleep (34.5%), and obese people (35.8%). Prevalence of arthritis was higher in females (30.0%), widowed (31.9%), those living in elderly care homes (27%), housewives (31.2%), economically dependent on family (29.8%), taking a non-vegetarian diet (26.0%), physically inactive (33.4%), having disturbed sleep (33.3%), having constipation (37.7%), and obese people (24.5%). Self-reported duration of illness, history of treatment, and disease control status (assessed by interviewers by checking the latest medical records and physical examination) are given in Table 2.

**Table 2:** Self-reported duration of illness, history of treatment, and disease control

Name of the disease	Total	Duration of illness in years, mean (SD)	Taking treatment		Disease under control	
			Yes, n (%)	No, n (%)	Yes, n (%)	No, n (%)
Hypertension	675	10.77 (8.11)	658 (97.5)	17 (2.5)	667 (98.8)	8 (1.2)
Diabetes mellitus	522	11.33 (8.4)	506 (96.9)	16 (3.1)	498 (95.4)	24 (4.6)
Heart disease	131	9.89 (9.03)	114 (87)	17 (13)	119 (90.8)	12 (9.2)
Dyslipidemia	117	8.09 (6.01)	107 (91.5)	10 (8.5)	111 (94.9)	6 (5.1)
Sensory impairment	168	8.02 (7.06)	84 (50)	84 (50)	113 (67.3)	55 (32.7)
Stroke	40	7.05 (5.4)	33 (82.5)	7 (17.5)	37 (92.5)	3 (7.5)
Psychiatric illness	11	11 (9.32)	7 (63.6)	4 (36.4)	8 (72.7)	3 (27.3)
Joint pain	369	7.7 (6.13)	283 (76.7)	86 (23.3)	279 (75.6)	90 (24.4)
Any other illness	284	10.65 (8.51)	171 (60.2)	113 (39.8)	175 (61.6)	109 (38.4)

Overall, 25.9% of elderly participants were apparently healthy, 34.1% had one NCD, 22.8% had two NCDs, and 17.2% had three or more NCDs. Overall, 74.1% (71.5% male and 77.0% female) participants had at least one NCD (one morbidity), and 40.0% (34.4% male and 46.1% female) people had two or more NCDs, called multimorbidity.

Table 3 shows adjusted odds ratios for each of the modifiable factors.

**Table 3:** Impact of modifiable lifestyle factors on NCDs. * = p<0.05; ** = p<0.01; *** = p<0.001 NCD, non-communicable disease

Predictors	Hypertension		Diabetes		Heart disease		High cholesterol		Sensory impairment		Stroke		Psychiatric illness		Joint pain	
	Odds ratios	CI	Odds ratios	CI	Odds Ratios	CI	Odds ratios	CI	Odds ratios	CI	Odds ratios	CI	Odds ratios	CI	Odds ratios	CI
(Intercept)	0.47^***^	0.40 – 0.55	0.37^***^	0.31 – 0.43	0.07^***^	0.05 – 0.09	0.04^***^	0.03 – 0.06	0.18^***^	0.11 – 0.29	0.06^***^	0.03 – 0.12	0.00^***^	0.00 – 0.01	0.31^***^	0.22 – 0.42
Sleep (disturbed)	1.89^***^	1.49 – 2.41	1.23	0.96 – 1.58	-	-	1.81^**^	1.18 – 2.74	-	-	-	-	0.23	0.01 – 1.34	1.57^**^	1.18 – 2.09
BMI Range (overweight and obese)	1.63^***^	1.33 – 1.99	1.43^***^	1.15 – 1.76	1.46^*^	1.02 – 2.12	1.50^*^	1.03 – 2.22	0.82	0.59 – 1.15	0.34^**^	0.16 – 0.68	-	-	1.31^*^	1.03 – 1.68
BMI range (underweight)	0.58	0.27 – 1.14	0.41^*^	0.16 – 0.93	0.63	0.10 – 2.13	0	0.00 – 41776.52	2.04	0.88 – 4.29	0.75	0.04 – 3.68	-	-	1.18	0.53 – 2.43
Total years of practicing physical activity	-	-	0.89^*^	0.80 – 0.99	-	-	1.33^***^	1.12 – 1.56	1.32^**^	1.11 – 1.57	-	-	1.63^*^	1.01 – 2.44	-	-
Habits total years	-	-	-	-	1.30^***^	1.12 – 1.50	-	-	1.28	0.94 – 1.72	-	-	-	-	-	-
Diet (non-veg)	-	-	-	-	-	-	1.44	0.98 – 2.12	-	-	1.63	0.86 – 3.14	-	-	1.44^**^	1.13 – 1.84
Physical activity duration (less than 30 mins)	-	-	-	-	-	-	-	-	0.53^*^	0.32 – 0.89	0.42^*^	0.19 – 0.95	-	-	0.38^***^	0.28 – 0.52
Physical activity duration (up to 1 hr)	-	-	-	-	-	-	-	-	0.56^*^	0.34 – 0.92	0.51	0.23 – 1.15	-	-	0.66^**^	0.48 – 0.89
Substance habit (having habits)	-	-	-	-	-	-	-	-	0.43	0.18 – 0.98	0.35	0.10 – 0.91	-	-	0.61^**^	0.44 – 0.83
Bowel (constipation)	-	-	-	-	-	-	-	-	2.21^***^	1.53 – 3.15	-	-	3.97^*^	0.96 – 14.18	1.72^***^	1.28 – 2.29
Observations	1671		1671		1671		1671		1671		1671		1671		1671	
R^2^ Tjur	0.031		0.015		0.01		0.02		0.026		0.012		0.007		0.066	

Among the factors, sleep has the highest odds ratio across hypertension, high cholesterol, and joint pain (AOR = 1.89, 95% CI: 1.49-2.41; AOR = 1.81, 95% CI: 1.18-2.74; AOR = 1.57, 95% CI: 1.18-2.09, respectively). It indicates that a person with disturbed sleep has increased risk of a specific disease by more than 80% compared to a person with proper sleep. Being overweight or obese also increases the risk for most common factors across all the diseases (hypertension: AOR = 1.63, 95% CI: 1.33-1.99; diabetes: AOR = 1.43, 95% CI: 1.15-1.76; heart disease: AOR = 1.46, 95% CI: 1.02-2.12; high cholesterol: AOR = 1.50, 95% CI: 1.03-2.22; stroke: AOR = 0.34, 95% CI: 0.16-0.68; joint pain: AOR = 1.57, 95% CI: 1.18-2.09). Obese people have 64% more risk of hypertension than people with normal BMI. Table 4 shows the adjusted odds ratios of the nonmodifiable factors.

**Table 4:** Impact of nonmodifiable lifestyle factors on NCDs. * = p<0.05; ** = p<0.01; *** = p<0.001

	Hypertension		Diabetes		Heart disease		High cholesterol		Sensory impairment		Stroke		Joint pain	
Predictors	Odds ratios	CI	Odds ratios	CI	Odds ratios	CI	Odds ratios	CI	Odds ratios	CI	Odds ratios	CI	Odds ratios	CI
(Intercept)	0.50 ^***^	0.40 – 0.62	0.55^***^	0.45 – 0.66	0.07^***^	0.04 – 0.10	0.02^***^	0.01 – 0.05	0.08^***^	0.05 – 0.11	0.01^***^	0.01 – 0.03	0.22^***^	0.14 – 0.36
Gender (female)	1.27	0.98 – 1.63	-	-	0.48^***^	0.32 – 0.73	1.60^*^	1.08 – 2.36	-	-	-	-	1.92^***^	1.44 – 2.56
Age range (>70)	1.37^**^	1.10 – 1.70	-	-	2.18^***^	1.43 – 3.45	1.46	0.98 – 2.23	2.34^***^	1.61 – 3.48	2.21^*^	1.05 – 5.21	1.47^**^	1.13 – 1.91
Marital status (divorced)	5.55^*^	1.55 – 25.97	-	-	4.39	0.64 – 18.38	-	-	-	-	-	-	3.63^*^	0.99 – 12.75
Marital status (never married)	1.94	0.90 – 4.18	-	-	1.88	0.29 – 7.04	-	-	-	-	-	-	2.38^*^	1.10 – 5.05
Marital status (widowed)	1.74^***^	1.33 – 2.28	-	-	1.72^*^	1.05 – 2.75	-	-	-	-	-	-	1.45^*^	1.08 – 1.94
Residing with (elderly care home)	0.71^*^	0.54 – 0.93	0.64^**^	0.49 – 0.83	0.53^*^	0.30 – 0.88	-	-	-	-	-	-	-	-
Residing with (separately)	0.72	0.47 – 1.09	0.78	0.49 – 1.19	0.7	0.30 – 1.43	-	-	-	-	-	-	-	-
Present work status (housewife)	1.11	0.84 – 1.47	-	-	-	-	-	-	-	-	0.95	0.43 – 1.94	-	-
Present work status (working full time)	0.64^*^	0.44 – 0.92	-	-	-	-	-	-	-	-	0	0.00 – 4022992370.15	-	-
Present work status (working part time)	0.67	0.45 – 1.00	-	-	-	-	-	-	-	-	1.6	0.53 – 3.96	-	-
Employment (private sector)	-	-	0.98	0.77 – 1.25	-	-	-	-	-	-	-	-	0.81	0.56 – 1.15
Employment (self-employed)	-	-	0.71^*^	0.53 – 0.96	-	-	-	-	-	-	-	-	0.63^*^	0.41 – 0.96
Education (above 12 years)	-	-	-	-	-	-	2.67	1.08 – 8.93	-	-	-	-	-	-
Education (two to six years)	-	-	-	-	-	-	1.95	0.77 – 6.57	-	-	-	-	-	-
Economic status (pension)	-	-	-	-	-	-	-	-	0.8	0.56 – 1.14	-	-	0.67^*^	0.47 – 0.94
Economic status (salary)	-	-	-	-	-	-	-	-	0.4	0.09 – 1.12	-	-	0.33^*^	0.11 – 0.77
Economic status (self-sustainable)	-	-	-	-	-	-	-	-	0.51^*^	0.29 – 0.84	-	-	0.65^*^	0.44 – 0.95
Observations	1671		1671		1671		1671		1671		1671		1671	
R^2^ Tjur	0.04		0.011		0.023		0.007		0.017		0.006		0.06	

Females have a higher risk for hypertension, high cholesterol, psychiatric illness, and joint pains than males. Except for psychiatric illness, those aged >70 years have a higher risk for NCDs than those aged <70 years. Widowed people are 1.5 times more at risk than people having a spouse. People whose marital status is “divorced” are the riskiest group.

Table 5 shows adjusted odds ratios of modifiable factors on morbidity across no disease versus one disease, no disease versus more than one disease, and one disease versus more than one disease.

**Table 5:** Impact of modifiable and non-modifiable factors on multimorbidity. * = p<0.05; ** = p<0.01; *** = p<0.001

Modifiable factors	No vs. one disease		One vs. two diseases		No vs. two diseases	
Predictors	Odds ratios	CI	Odds ratios	CI	Odds ratios	CI
(Intercept)	1.25	0.89 – 1.78	0.92	0.77 – 1.11	1.21	0.85 – 1.75
Physical activity duration (less than 30 min.)	0.69^*^	0.48 – 1.00	-	-	0.62^**^	0.44 – 0.88
physical activity duration (up to 1 hr)	0.97	0.66 – 1.41	-	-	0.73	0.50 – 1.06
Sleep (disturbed)	1.39	0.96 – 2.02	1.52^**^	1.14 – 2.03	2.12^***^	1.48 – 3.06
Bowel (constipation)	1.44	1.00 – 2.10	1.34^*^	1.00 – 1.80	1.92^***^	1.33 – 2.79
BMI Range (overweight)	1.39^*^	1.08 – 1.81	1.33^*^	1.05 – 1.67	1.84^***^	1.42 – 2.38
BMI range (underweight)	0.59	0.26 – 1.29	1.31	0.60 – 2.99	0.69	0.32 – 1.47
Habits, total years	-	-	1.02^*^	1.00 – 1.04	-	-
Substance habit (having habits)	-	-	0.43^**^	0.25 – 0.74	0.77	0.55 – 1.07
Diet (non-veg)	-	-	-	-	1.22	0.94 – 1.59
Observations	1003		1239		1100	
R^2^ Tjur	0.028		0.024		0.074	
Nonmodifiable factors						
(Intercept)	1.28	0.77 – 2.12	1.47^**^	1.12 – 1.93	0.96	0.49 – 1.88
Gender (female)	1.26	0.93 – 1.71	-	-	1.45^*^	1.07 – 1.97
Age range (>70)	1.39^*^	1.07 – 1.81	1.27	0.98 – 1.63	1.81^***^	1.39 – 2.36
Employment (private sector)	0.55^**^	0.38 – 0.79	-	-	0.59^**^	0.41 – 0.85
Employment (self-employed)	0.56^**^	0.37 – 0.85	-	-	0.54^**^	0.35 – 0.82
Economic status (pension)	0.87	0.59 – 1.28	0.63^**^	0.48 – 0.83	0.59^**^	0.41 – 0.84
Economic status (salary)	2.99^**^	1.53 – 6.12	0.16^***^	0.08 – 0.32	0.51	0.21 – 1.21
Economic status (self-sustainable)	2.46^***^	1.62 – 3.75	0.35^***^	0.25 – 0.49	0.99	0.64 – 1.52
Marital status (divorced)	-	-	2.16	0.56 – 10.40	2.7	0.58 – 19.20
Marital status (never married)	-	-	4.88^**^	1.83 – 15.45	1.4	0.60 – 3.55
Marital status (widowed)	-	-	2.53^***^	1.83 – 3.51	1.74^**^	1.25 – 2.44
Residing with (elderly care home)	-	-	0.59^***^	0.43 – 0.80	-	-
Residing with (separately)	-	-	0.97	0.59 – 1.61	-	-
Education (above 12 years)	-	-	-	-	1.6	0.94 – 2.73
Education (two to six years)	-	-	-	-	1.72^*^	1.03 – 2.88
Observations	1003		1239		1100	
R^2^ Tjur	0.047		0.083		0.065	

Among the correlates, obesity is the common risk factor across all the morbidity segments. Disturbed sleep increases the risk two times for multimorbidity (AOR = 2.12, 95% CI: 1.48-3.06). Constipation is positively associated with multimorbidity compared to one disease and no disease (AOR = 1.34, 95% CI: 1.00-1.80; AOR = 1.92, 95% CI: 1.33-2.79). Substance habit and prolonged usage increase the risk for multimorbidity. Physical activity duration of less than 30-minute duration increased the risk of having more than one disease (AOR = 0.64, 95% CI: 0.44-0.88). As the age increased, the risk of having multiple diseases doubled (AOR = 1.39, 95% CI: 1.07-1.81).

## Discussion

This is a cross-sectional survey involving a comprehensive sample of urban Indian elderly residents. The study revealed that about 71% of the survey participants had one chronic NCD, and 40.0% elderly had multimorbidity (≥ two NCDs). These findings are similar to the National Chinese Prevalence study, and the most prevalent NCDs were hypertension followed by diabetes [18]. Compared to an earlier study, BKPAI-2011, in India and South African elderly survey, the self-reported prevalence of at least one chronic NCD and multimorbidity prevalence is higher in the present study [19,20]. In the SAGE study, the prevalence of self-reported NCDs in urban elderly residents was 24.7% for hypertension and 18.1% for arthritis. In contrast, in the present survey, prevalence of hypertension is 40.4% and that of arthritis is 22.1%. This indicates that there are increasing trends in the prevalence of hypertension and arthritis. The BKPAI-2011 study reported that the most chronic NCD experienced by the urban elderly was arthritis. In the present study, hypertension is highly experienced, followed by diabetes, and has a higher prevalence than in the BKPAI-2011 study. This indicates that these NCDs are higher in urban Indian elderly residents. The survey found that the prevalence of NCDs increases with increasing age. It is also corroborating with preliminary evidence [21,22]. NCDs contributed to the disease burden in India, and this transition happened from 1990 to 2010 [21]. Furthermore, the disease burden is increasing due to NCDs. Old-age people had a higher prevalence of these NCDs compared to an earlier decade.

This survey also has shown that multimorbidity differed significantly by gender, age range, present work status, and economic status. Multimorbidity was higher in females, whereas having one morbidity was higher in males. The age range of 60 to 70 years has a higher prevalence of multimorbidity. In the BKPAI-2011 survey, multimorbidity among the elderly was 23.6%, whereas, in the current study, it is 40%. It shows that multimorbidity is increasing. Another survey of the Chinese urban elderly has shown that multimorbidity is increasing among the elderly and has a similar prevalence (45%). The most common conditions were hypertension and arthritis [23]. Similar trends were reported in the Canadian study for multimorbidity [24].

Health determinant-wise, multimorbidity is higher among the surveyed people who have disturbed sleep, are physically inactive, have substance abuse habits, and have constipation. These findings align with previous reports on the association between lifestyle factors and multimorbidity [25-27]. It indicates that individual health risk factors related to lifestyle have a significant role in multimorbidity among the elderly. Apart from the known risk factors for multimorbidity, this study shows that bowel clearance is a novel health determinant. Worldwide, multimorbidity is increasing among the elderly. It results in polypharmacy and also disabilities. It leads to more challenges in managing health. Implementation of an integrative approach and guidelines such as the National Institute for Health and Care Excellence of England for elderly care is the need of the hour [17]. The National Programme for Health Care of the Elderly (NPHCE) in India has been envisaged to provide promotional, preventive, curative, and rehabilitative services in an integrated manner for the elderly in various government health facilities. However, the challenges in implementation include providing acute services, establishing infrastructure, and ensuring enabling environment for healthy ageing [28]. Only recently was a nationally representative survey of older persons in India undertaken and released this year, 2022 [29].

Limitations

In this study, NCDs are considered based on self-reported and previous diagnoses. However, self-reports cannot capture undiagnosed conditions, which may be a significant proportion of people with NCDs [30]. Hence, the bias of underreporting cannot be ruled out. This study was conducted among the elderly with an age range of 60 to 80 years; therefore, the entire elderly cohort was not represented. People aged 60 to 80 years have a higher prevalence of NCDs than those over 80 years [8]. Also, few participants belonged to old homes, which may influence the overall prevalence. The study also reports limitations of selection (selection of cities and participants), information, and recall bias.

Strengths

This study is one of few studies conducted among urban elderly with a large sample size and geographic representation in India, apart from national surveys. This study's findings will help policymakers take appropriate public health actions.

## Conclusions

We conclude that the prevalence of hypertension, diabetes mellitus, joint pain, and multimorbidity is high among urban elderly in India. Due importance should be given to a healthy lifestyle to reduce the burden of NCDs and multimorbidity among urban elderly. Developing holistic health policies and their implementation is crucial for ensuring healthy aging.
